# Exploring Evolutionary and Transmission Dynamics of HIV Epidemic in Serbia: Bridging Socio-Demographic With Phylogenetic Approach

**DOI:** 10.3389/fmicb.2019.00287

**Published:** 2019-02-25

**Authors:** Luka Jovanović, Marina Šiljić, Valentina Ćirković, Dubravka Salemović, Ivana Pešić-Pavlović, Marija Todorović, Jovan Ranin, Djordje Jevtović, Maja Stanojević

**Affiliations:** ^1^Institute of Microbiology and Immunology, Faculty of Medicine, University of Belgrade, Belgrade, Serbia; ^2^Infectious and Tropical Diseases University Hospital, Clinical Centre of Serbia, Belgrade, Serbia; ^3^Virology Laboratory, Microbiology Department, Clinical Centre of Serbia, Belgrade, Serbia

**Keywords:** HIV-1, subtype B, MSM, transmission clusters, model, phylodynamics, latent class analysis, Serbia

## Abstract

Previous molecular studies of Serbian HIV epidemic identified the dominance of subtype B and presence of clusters related HIV-1 transmission, in particular among men who have sex with men (MSM). In order to get a deeper understanding of the complexities of HIV sub-epidemics in Serbia, epidemic trends, temporal origin and phylodynamic characteristics in general population and subpopulations were analyzed by means of mathematical modeling, phylogenetic analysis and latent class analysis (LCA). Fitting of the logistic curve of trends for a cumulative annual number of new HIV cases in 1984–2016, in general population and MSM transmission group, was performed. Both datasets fitted the logistic growth model, showing the early exponential phase of the growth curve. According to the suggested model, in the year 2030, the number of newly diagnosed HIV cases in Serbia will continue to grow, in particular in the MSM transmission group. Further, a detailed phylogenetic analysis was performed on 385 sequences from the period 1997–2015. Identification of transmission clusters, estimation of population growth (Ne), of the effective reproductive number (Re) and time of the most recent common ancestor (tMRCA) were estimated employing Bayesian and maximum likelihood methods. A substantial proportion of 53% of subtype B sequences was found within transmission clusters/network. Phylodynamic analysis revealed Re over one during the whole period investigated, with the steepest slopes and a recent tMRCA for MSM transmission group subtype B clades, in line with a growing trend in the number of transmissions in years approaching the end of the study period. Contrary, heterosexual clades in both studied subtypes – B and C – showed modest growth and stagnation. LCA analysis identified five latent classes, with transmission clusters dominantly present in 2/5 classes, linked to MSM transmission living in the capital city and with the high prevalence of co-infection with HBV and/or other STIs.Presented findings imply that HIV epidemic in Serbia is still in the exponential growth phase, in particular, related to the MSM transmission, with estimated steep growth curve until 2030. The obtained results imply that an average new HIV patient in Serbia is a young man with concomitant sexually transmitted infection.

## Introduction

The pandemic of human immunodeficiency virus (HIV) infection has developed in numerous heterogeneous sub-epidemics worldwide, substantially influenced by patterns of human migration and globalization. Similar to what happened worldwide, Serbian HIV epidemic has evolved through several phases. Serbia is a low HIV-1 prevalence country. According to the national surveillance data, since the first HIV cases emerged in 1985 until the end of 2017, over 3,500 people in Serbia were living with HIV/AIDS, without substantial reduction in the yearly number of newly diagnosed cases^1^. In the beginning, the HIV epidemic in Serbia spread mostly among people who inject drugs (PWID), until the mid-1990s, when the heterosexual transmission route became more prevalent. In recent years another shift in the dominant transmission route was observed, leading to the current situation where HIV epidemic is driven by transmission among men having sex with men (MSM), comprising the majority of new HIV diagnoses, with the yet ongoing presence of heterosexual transmission.

Previous molecular studies of Serbian HIV epidemic identified subtype B as the dominant HIV-1 type, present in over 90% of individuals diagnosed between 1997 and 2011 (Stanojevic et al., 2002, 2014; Siljic et al., 2013; Magiorkinis et al., 2016). Clusters related HIV-1 transmission was observed, in particular among MSM transmission group and linked to high prevalence of concomitant sexually transmitted infections (STIs) (Siljic et al., 2013). This rapid expansion of HIV-1 infection among MSM transmission group and high prevalence of reported STIs underlined the need for detailed molecular investigation of the network structure and dynamics of viral transmission in this and other groups.

In order to get a deeper understanding of the complexities of HIV sub-epidemics in Serbia, epidemic trends, temporal origin and phylodynamic characteristics in general population and subpopulations were analyzed by means of mathematical modeling, phylogenetic analysis, and latent class analysis (LCA).

## Materials and Methods

### Logistic Growth Modeling

Logistic growth modeling was performed based on data about new HIV cases, as well as the basic related demographic data in the period 1984–2016, available from the Institute of Public Health of Serbia “Dr. Milan Jovanovic Batut” and the annual HIV reports of the European Centre for Disease Control (see footnote 1; ECDC, 2017).

Fitting of the logistic curve of trends for a cumulative annual number of new HIV cases in the period 1984–2016, in general population, heterosexual transmission group, PWID transmission group and MSM transmission group, was performed with the software NLREG, v 6.6^2^. The logistic regression model assumes the existence of a maximum population size which a given environment can accommodate (carrying capacity alias K) and a biphasic growth rate creating two stages of growth: (i) an early exponential phase and (ii) a late phase of the plateau (Vandermeer, 2010). In the context of an HIV epidemic the exponential phase of logistic growth could illustrate the absence of large scale preventive measures or the presence of inefficient preventive policy (Fonseca and Bastos, 2007). The following logistic growth model was used: *y* = *K*/(1 + exp^∗^(*a* + *b*^∗^*x*)), where *K* represents carrying capacity, *a* and *b* represent parameters that shape and scale the function (Sherrod, 2010). NLREG provides an estimation of parameters *K*, *a* and *b* with the strongest statistical back-up. It also provides several diagnostic statistical tests and variables which describe the statistical strength of the proposed model such as (i) probability t (prob. t) is a probability that the estimated parameter (*a*, *b* and *K*) is 0; prob. t less than 0.05 is considered as a good estimation of parameter significance for the model; (ii) probability f (prob. f) is the probability that all of the regression parameters are 0, and it ranges from 0 to 1; prob. f less than 0.05 illustrates an overall statistical significance of the model. The model obtained was used to predict further trends of HIV epidemic in general population and by transmission groups (MSM, PWID, heterosexual transmission) for the period 2017–2030. This analysis provided a general overview of the epidemiological background for further transmission clusters analysis.

### Phylogenetic Analyses

#### Study Population and Sequence Dataset

The earliest viral sequences from Serbian epidemic date from 1997. For the time period considered, 1997 to 2011, data and sequences were gathered from previous molecular studies of HIV epidemic in Serbia. Complete dataset of these sequences is available at the NCBI database, accession numbers are given in Supplementary File 1). Additionally, this study included blood samples collected from 2012 to 2015, from consenting HIV seropositive adults, both therapy naïve and therapy experienced, followed at the Centre for HIV/AIDS, University Hospital for Infectious and Tropical Diseases in Belgrade. Blood samples from HIV-infected patients were sent for drug resistance testing as part of patients’ routine follow-up. The study was approved by the Ethical Committee of the University of Belgrade Faculty of Medicine 29/V-11.

In brief, HIV-1 genomic RNA was extracted from 140 μL of stored plasma specimens using the QIAmp Viral RNA Mini kit (Qiagen, Hilden, Germany). Reverse transcription and nested PCR amplification of partial pol gene were performed using One Step RNA PCR Kit (Qiagen, Hilden, Germany), Thermo scientific dreamTaq PCR master mix (2X) (Applied Biosystems, Foster City, CA, United States) and HIV-1 specific primer sets (Snoeck et al., 2005). Upon successful amplification and purification with MinElute Purification Kit (Qiagen, Hilden, Germany), according to the manufacturer’s instruction, PCR products were subjected to direct sequencing of both the sense (forward) and antisense strands (reverse) by BigDye^®^Terminator v3.1 (Applied Biosystems, Foster City, CA, United States).

Obtained sequences were visually inspected, manually edited and then assembled with SeqScape HIV-1 Genotyping System Software v 2.5 (Applied Biosystems, Foster City, CA, United States). Sequences generated in this research were deposited in the NCBI database (accession numbers are shown in Supplementary File 1).

Reference sequences of different subtypes, known to be present locally in Serbia and in the Balkan region, encompassing subtypes B, C, G, A, F, as well as circulating recombinant forms (CRFs), CRF01_AE and CRF02-AG, were downloaded from the Los Alamos database^3^. Additionally, NCBI BLAST search was performed for each sequence found to belong to a transmission cluster (according to criteria described in detail further). For each query sequence, five sequences were included in the tree reconstruction based on the highest similarity score as obtained by NCBI BLAST search. In total, 175 sequences were included in the analyses after BLAST search. Furthermore, a group of control/background sequences was also included, in the context of the origin and spread of HIV from the isolates of North America, West Europe, and Balkan, with the clear defined subtype and the time of sampling available at the NCBI database (accession numbers are shown in Supplementary File 1).

Data on clinical characteristics (CDC stage, baseline CD4 count, coinfections and other sexually transmitted illnesses), epidemiological background (age, gender, date of diagnosis, residence and level of education) and risk for acquiring HIV infection (most probable HIV transmission route) were used as a covariate of transmission analysis.

#### HIV Subtyping

Subtyping of all sequences included in the study was firstly performed using the REGA HIV-1 subtyping tool version 3 (REGA v3)^4^ and the Los Alamos HIV database (see footnote 3). The Rega subtyping tool is based on phylogenetic analysis in order to take into account the epidemiological and evolutionary relationships among subtypes (Robertson et al., 2000). Subtyping of all pol gene sequences included in this research was further performed by construction of ML and NJ phylogenetic tree, under appropriate models, using the PAUP and MEGA software, with reference sequences of different subtypes, downloaded from HIV-1 Los Alamos Database (LANL ^5^). Sequences that were not unambiguously classified by REGA subtyping tool or that were assigned to a different subtype from the one specified in the Los Alamos database were removed from the data set.

#### Phylogenetic Trees Reconstruction

To eliminate the influence of antiretroviral drug selective pressure on viral evolution, we made a codon-stripped sequence alignment by removing drug resistance-associated codons identified by the Stanford Drug Resistance Database^6^ (Wensing et al., 2017). Analysis was also performed without codons removing. Both analyses gave congruent results with no evidence of the selection pressure of antiretroviral drugs to the clustering patterns. Sequences were aligned using the Clustal W algorithm implemented in MEGA v 6^7^ and were manually edited.

The Bayesian phylogenetic tree was reconstructed through Bayesian inference of phylogeny by using MrBayes software (Ronquist and Huelsenbeck, 2003). A Markov Chain Monte Carlo (MCMC) search was made for 10 × 106 generations using tree sampling every 100th generation and a burn-in fraction of 20%. Statistical support for specific clades was obtained by calculating the posterior probability of each monophyletic clade, and a posterior consensus tree was generated after a 25% burn-in.

Maximum likelihood (ML) tree reconstruction using PAUP was performed under GTR model with discrete gamma rates heterogeneity among sites and the proportion of invariable sites, as selected by jModelTest (Swofford, 1999; Guindon and Gascuel, 2003; Posada, 2009).

Phylogenetic analyses were conducted: (i) to confirm subtype assignment performed with REGA subtyping tool (ii) to identify the presence of local transmission networks (phylogenetic clusters). and (iii) to perform in-depth reconstruction of demographic and transmission history of the HIV epidemic in Serbia.

#### Analysis of Transmission Clusters

A set of analyses was performed for a definition of HIV transmission clusters, following the strategy of “statistical support of clades plus similarity” that proved to be suitable in our previous investigation of transmission chains in Serbia (Siljic et al., 2013, 2017). Evidence of transmission cluster was characterized by two criteria sets: according to the first set of criteria, transmission clusters were assigned as those monophyletic phylogenetic clades consisting of three or more sequences, fulfilling the conditions of genetic distance of 1.5% or less, with minimal bootstrap support of 90%, and the Bayesian posterior probability of higher than 0.9. Genetic distances were first determined using PAUP^∗^ version 4.0 software, based on ML analysis and under general time reversible model (GTR) with gamma distribution and proportion of invariable sites (Swofford, 1999; Guindon and Gascuel, 2003), selected by jModelTest statistical analyses (Posada, 2009). Branch supports were estimated using bootstrap analysis with 1,000 replicates. Phylogenetic trees were visualized in FigTree program version 1.3.1^8^. Second criteria sets, including those clades with bootstrap support over 75% and with genetic distance of less than 4%, were further analyzed.

For each sequence found within a cluster according to the second criteria set, 5 most similar sequences were identified using BLAST analysis^9^ and included in tree reconstruction – subsistence of the initial local clusters after inclusion of BLAST identified sequences in the tree reconstruction led to their designation as true transmission clusters.

#### Bayesian Molecular-Evolution Analyses

##### Timed evolutionary histories, demographic history, and reproductive effective number estimates

Subtype B MSM transmission network and the two most expanded MSM transmission clusters (composed of 15 and 11 sequences) identified in the previous analyses, were subjected to detailed temporal and phylodynamic reconstruction aimed to infer demographic and evolutionary histories of these monophyletic groups.

Additionally, two heterosexual monophyletic clades were analyzed, one each of subtypes B and C, in order to analyze these sub-epidemics. The studied subtype C clade was supported by 100% value of bootstrap support and composed of sequences from heterosexuals only, whereas for subtype B, phylodynamic analyses were performed on a monophyletic clade, with lower than predefined bootstrap value, but composed of sequences dominantly isolated from heterosexuals. Moreover, as a method of validation against the possible bias due to low bootstrap support seen in subtype B heterosexual clade, an additional monophyletic clade with similar bootstrap support, comprised of sequences from MSM transmission group, was chosen to be analyzed. The results of these analyses and detailed methods are presented in the Supplementary File 2.

Phylodynamic analyses in this research were performed as follows: estimations of evolutionary and demographic parameters were performed in BEAST software package v 1.7.5 and encompassed selection for site model, demographic model, and clock model prior to run MCMC chains. In the first step, the Bayesian skyline plot method (Drummond et al., 2005, 2012), was used to estimate effective population size (Ne), and to run a demographic analysis in Tracer v1.6^10^. In the second step, as for the coalescent priors, two different demographic models were compared: exponential and logistic growth; estimates of the population growth rate were then obtained under the model that provided the best fit to the demographic signal in each data set. The best fit coalescent model was chosen by means of a Bayes factor (BF), using marginal likelihoods, determined by Tracer version 1.6. Each analysis was performed under GTR+G+I, as the best fit nucleotide substitution model, and uncorrelated lognormal relaxed clock, as shown to be the most suitable for HIV datasets. MCMC chains were run for 5 × 107 generations for each data set, with a burn-in of 10%. BEAST output was analyzed using TRACER v1.6, with uncertainty in parameter estimates reflected as the 95% highest probability density (HPD). Convergence of parameters was assessed through the ESS after excluding an initial 10% for each run. All parameter estimates for each run showed ESS values >200. A graphical representation of the effective number of infections through time was generated by using TRACER v1.6.

In order to estimate the time of the most recent common ancestors for the selected clades, molecular clock analysis was performed using a Bayesian MCMC coalescent method, as implemented in BEAST v1.8.1 (Drummond et al., 2003; Drummond and Rambaut, 2007). We used GTR nucleotide substitution model with six category gamma distributed rate variation among sites and two partitions in the codon positions (the best fitting model for all three datasets according to jModelTest). Bayes factor analysis, performed in Tracer v.1.5, showed that the lognormal relaxed clock with the Bayesian Skyline was the best model, as indicated by a log Bayes Factor > 10 according to Tracer v1.5^11^ – evidence of very strong statistical support (Suchard et al., 2001). Convergence of the Markov chain was assessed by program Tracer v 1.5^12^ calculating the effective sample size (ESS) for each parameter.

The last part of phylodynamic analyses was the estimation of the effective reproductive number conducted in BEAST2 v 2.1.3 The analysis employed a general time-reversible substitution model with a gamma-distributed rate variation and proportion of invariant sites (GTR+Γ4+I), an uncorrelated lognormal relaxed molecular clock model (Drummond et al., 2006), and a Birth-Death Skyline Serial model (BDSKY) as a model for viral transmission (Stadler et al., 2012, 2013). The following prior distribution of the BDSKY model parameters was used: LogNorm (0; 10) for effective reproductive number R and LogNorm (0; 10) for the rate of becoming non-infectious δ. The BEAST2 analyses were run until all relevant parameters converged, with 20% of the MCMC chains discarded as burn-in. Statistical confidence was represented by values for the 95% HPD. To generate the log file, five independent MCMC runs of 2 × 108 chain length were combined with Log Combiner (Drummond et al., 2012). Sampling dates were used to infer the tree height and internal node ages in the maximum clade credibility (MCC) time-trees using BEAST2 (Bouckaert et al., 2014). Similarly to the log file, the time-trees from five independent runs of 2 × 108 chain length were combined with Log Combiner (Drummond et al., 2012) with 20% of the MCMC chains discarded as burn-in.

### Latent Class Analysis

Latent class analysis is a statistical tool that explores underlying patterns of covariance in the data structure to identify subgroups or ‘discrete classes’ of participants’ epidemiological, behavioral and clinical profiles. Class membership is inferred based on an individual’s pattern of responses across a set of variables but not directly observed and therefore classes are considered to be latent. In this study, LCA was conducted to examine and identify participants risk profiles regarding 11 types of latent class indicators (categorical latent variable). Latent class indicators included: gender, transmission risk, age, residence, education level, coinfections with hepatitis B virus (HBV) and hepatitis C virus (HCV), presence of other STIs, CDC stage at the time of diagnosis, time-period of diagnosis and inclusion of sequence within clusters/network. These classification variables were selected to represent a range of established HIV risk factors in order to identify comprehensive HIV profiles, based on clinical, epidemiological, demographic and phylogenetic data. The covariates were separated into ordinal categories where each category was assigned a nominal value of 1 to 4.

Latent class analysis was performed in R software with polytomy variable LCA (poLCA) software package. PoLCA is a user-friendly package for the estimation of latent class models and latent class regression models in R available from the Comprehensive R Archive Network^13^ (Linzer and Lewis, 2011; Schreiber, 2017). Based on the fact that this algorithm may locate a local, rather than global maximum, nrep was set to 10, which increased the probability that the global maximum log-likelihood would be located. Parameters used to select the optimal number of latent classes in LCA included the Akaike information criteria (AIC) and the Bayesian information criteria (BIC), two most widely used parsimony measures (Akaike, 1973; Schwarz, 1978). We began with a 1-class model and increased the number of classes in each subsequent model seeking to minimize both the BIC and the AIC value before these values increased with the addition of another class.

### Statistical Analysis

Results were analyzed by standard statistical tests. Categorical data were compared using the chi-square test and Fisher’s exact test. Age and time of diagnosis of patients within network and clusters were statistically analyzed using chi-square test available at https://www.graphpad.com/quickcalcs/chisquared1.cfm.

## Results

### Logistic Growth Modeling

**FIGURE 1:**
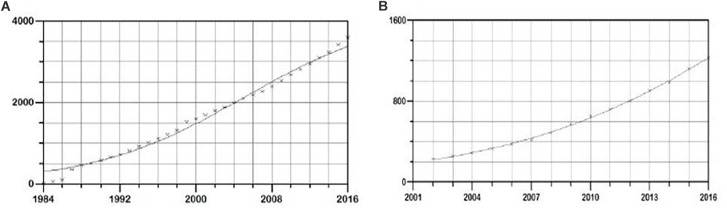
Logistic growth model of new HIV infections in **(A)** general population and **(B)** MSM population in Serbia, in the period 1985–2016. On *y*-axis cumulative number of new HIV cases is shown, and on the *x* time period in years is shown.

In the period 1984–2016, the total cumulative number of new HIV cases in Serbia was 3590. The applied logistic growth model fitted accurately the growth trend in this time period (Figure 1A). Software estimated parameters *K*, *a*, and *b* equaling 4227 ± 271, 2.61 ± 0.07, -0.12 ± 0.008, respectively, with prob. t parameter 0.00001. Overall statistical significance was illustrated with prob. f = 0.00001.

Since data on transmission routes became available starting from 2003, the incidence of new HIV infections through MSM, PWID, and heterosexual transmission route in the period 2003–2016 was used in the analysis.

In this time period, a total of 2,785 new cases of HIV in MSM were detected. Again, the logistic growth model fitted the growth trend accurately (Figure 1B). The estimated function parameters *K*, *a*, and *b* were found to be 3377 ± 455, 3.12 ± 0.11, -0.15 ± 0.005, respectively, with prob. t = 0.00001. Overall statistical significance illustrated with prob. f 0.00001 showed strong statistical support for the proposed model. In the same time period, a total of 979 and 850 new cases of HIV in PWID and heterosexuals were found, respectively. The estimated function parameters *K*, *a* and *b* were 1018 ± 7, 1.74 ± 0.04, -0.8 ± 0.1 with prob. t = 0.00001 and with strong statistical significance of the model (prob. f = 0.00001) in PWID transmission group and 1576 ± 176, 1.06 ± 0.14, -0.08 ± 0.0059 with prob. t = 0.00001 and strong statistical support of the model (prob. f = 0.00001) in the heterosexual transmission group.

**FIGURE 2:**
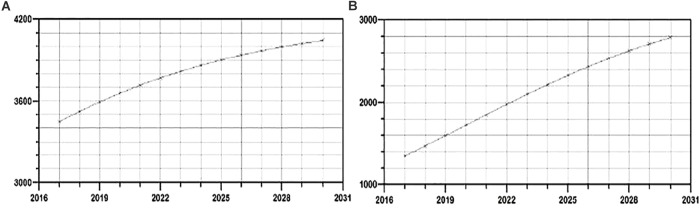
Evaluation of the growth trends for the period 2017–2030 of new HIV infections in **(A)** general and **(B)** MSM population in Serbia.

The fitted model was used to create trends of HIV epidemic for the period 2017–2030 in general population and by transmission groups. The modeled trend predicts approaching plateau in new HIV infections number by 2030 in general population (Figure 2A), whereas in MSM transmission group an exponential-like curve with no signs of a plateau in the studied time period was obtained (Figure 2B). Plateaued growth was observed in both PWID transmission group and heterosexual group (Supplementary Figures 4, 5).

### Phylogenetic Analyses

Phylogenetic analyses in the present study included a total of 385 local sequences, collected between 1997 and 2015 from both treatment naive and treatment experienced HIV-1 positive subjects. This pool represents around 11% of the total number of registered HIV+ individuals in Serbia in the same time period. Of these, the majority were male (81%), with a mean age of 37.5 years and reporting MSM contact (83.4%) as the most probable route of HIV acquisition. Epidemiological, demographic and clinical data are shown in Table 1.

**Table 1:** General epidemiological, demographic, and clinical data.

*Gender*	*N*	%
Male	312	81
Female	73	19
*Transmission route*		
MSM	302	78.4
Heterosexual	68	17.6
PWID	15	3.8
Unknown		
*Place of residence*		
Urban area	315	81.8
Rural	70	18.8
Unknown		
*CDC disease stage*		
A	175	45.5
B	93	24.1
C	117	30.4
Unknown		
*OSTI*		
Positive	87	22.6
Negative	298	77.4
Unknown		
*Median age*		
Male	34.2	
Female	39.5	


*MSM, men who have sex with men; PWID, people who inject drugs; CDC, Center for Disease Control; OSTI, other sexually transmitted infections.*

In total, subtype B was confirmed to be the predominant one, accounting for 90.9% of cases (350/385), while the prevalence of non-B subtypes was 9.1% (35/385). Among non-B subtypes, subtype C was found with the highest prevalence, in 3.1% of samples (12/385), followed by subtype G in 2.1% (8/385) and subtypes A in 1.3 % (8/385). CRFs were detected in 2.6% (10/385) of the collected samples.

**FIGURE 3:**
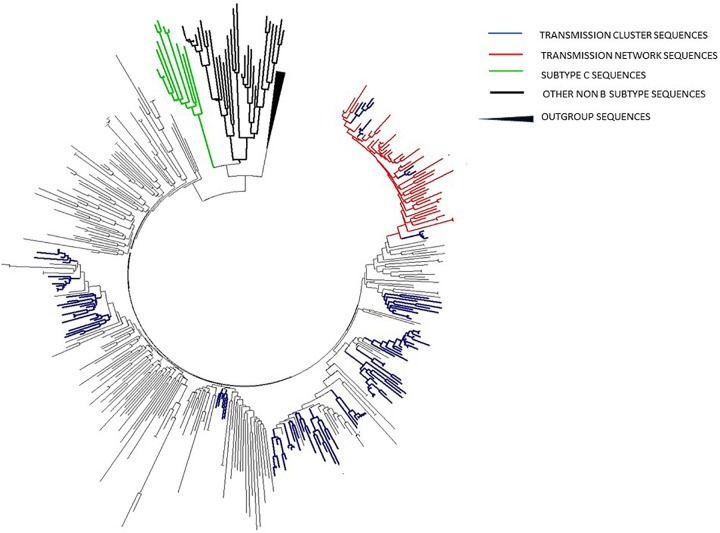
ML phylogenetic tree constructed in MEGA software V6.1 including all sequences analyzed in the current study together with reference sequences and background sequences from the NCBI database.

In phylogenetic trees reconstruction, both ML and Bayesian approach gave congruent results, revealing that 27.7% (107/385) of sequences were grouped within 19 transmission clusters, accomplishing predefined strict criteria (including nt distance of ≤1.5%), all within subtype B (Figure 3; labeled in dark blue). Moreover, 49 additional sequences were found grouped within a transmission network, a clade with high bootstrap support as well as posterior probability value but with a genetic distance of 8.2%, higher than the predefined cut off of 1.5% (Figure 3; labeled in red). This network contained sequences sampled in the time frame of 19 years and comprised a total of 63 sequences, encompassing thus 16.3% (63/385) of the total number of studied sequences. Taken together, the number of sequences within transmission clusters/network totaled to 205/385 (53%). Importantly, the majority of clusters, 15/19 (79%) were made solely of MSM transmission group sequences; with only 4/19 (21%) transmission clusters containing sequences of heterosexuals. Sequences contributing to transmission clusters network were from individuals of a mean age of 35.3 years (*SD* = 4.8) and were mostly men (*N* = 121/147; 82.3%). Specifically, the large transmission network encompassed sequences from older patients of a mean age of 39.4 years, in contrast to MSM clusters with members mean age of 32.6 (*SD* = 3.2) with particular emphasis on the most expanded transmission cluster of 15 sequences with a mean age of 29.2 (*SD* = 2.8). Statistical evaluation using the chi-square test revealed that a significantly lower number of patients found in MSM clusters were diagnosed prior to 2006 (*p* = 0.0184) while a significantly higher number of them was younger than 35 (*p* = 0.0388). Among non-B clades, subtype C cluster contained sequences with the smallest nt distance (6.5%) and from heterosexual patients, with bootstrap support of 100; therefore we used this clade for further phylodynamic analysis. The observed subtype C genetic distance exceeded the pre-defined criteria for transmission clusters, hence it was not classified as one, however, downstream phylodynamic analyses were still performed, in order to be able to make comparisons to the relevant subtype B clades (Figure 3; labeled in green).

The time of the most recent common ancestor for transmission network (composed of 63 sequences) was dated to the beginning of nineties (1993 HPD: 1988–1995), while for the two MSM clusters of 15 and 11 sequences it was dated to 2008 (HPD: 2003–2013) and 2005 (HPD: 2000–2010), respectively. Dating analysis of two heterosexual clades suggested that the most recent common ancestor of the subtype B and subtype C clusters was present approximately in the 1990 (HPD: 1984–1996) and 1989 (HPD: 1985–1993), respectively.

**FIGURE 4:**
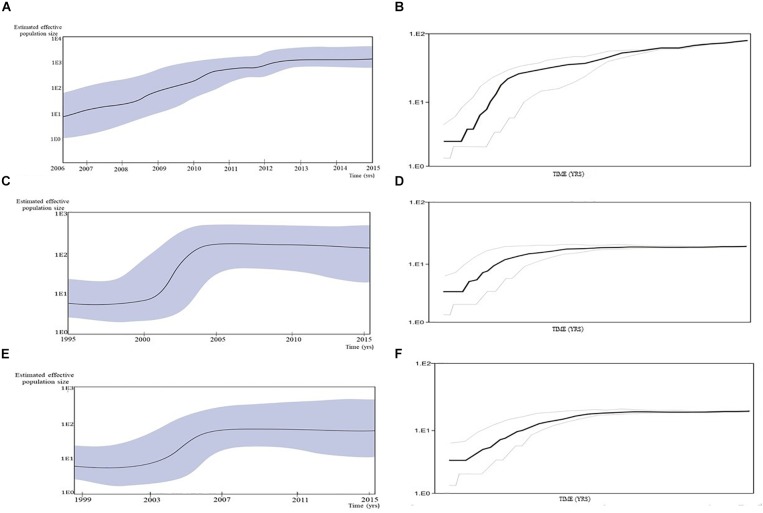
Population growth and the cumulative number of lineages (infections) in a logarithmic scale over time for the **(A,B)** transmission cluster of 15 sequences; **(C,D)** transmission network; **(E,F)** transmission cluster of 11 sequences. The median estimate of the effective number of infections (solid line) and 95% confidence limits of the estimate (dashed lines) are shown in each graphic.

**FIGURE 5:**
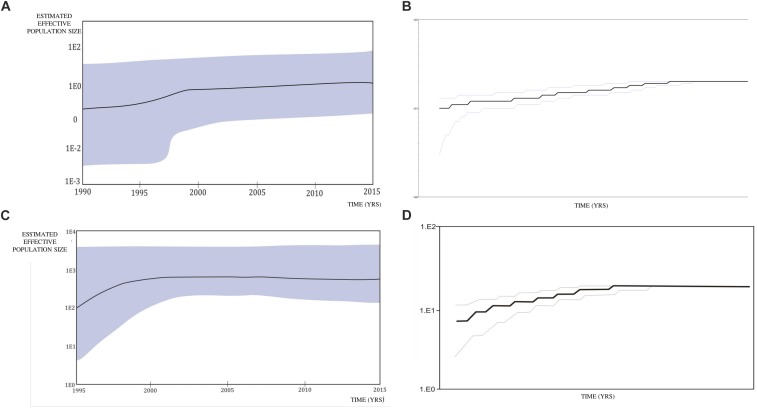
Bayesian Skyline plot and logistic growth analyses performed in BEAST software package v1.7.5 presenting population growth and the cumulative number of lineages (infections) in a logarithmic scale over time for **(A)** and **(B)** the subtype B clade composed of sequences from heterosexuals; **(C)** and **(D)** the subtype C clade composed of sequences from heterosexuals.

A Bayesian Skyline plot and logistic growth analyses were performed for three MSM clades and two heterosexual clades, of subtype B and subtype C sequences. The highest exponential growth in almost 2 logs was identified for the most expanded transmission cluster, which was also the most recent one, in the whole analyzed period (Figure 4A,B). A demographic reconstruction of the transmission network and transmission cluster of 11 sequences showed slightly lower population growth, still with initial exponential growth in over one log which stabilized in the mid-decades of the 2000s, as reflected in a stationary phase approaching the present (Figure 4C–F). Bayesian skyline reconstruction and logistic growth analyses for the heterosexual clade of subtype B showed an initial increase of estimated effective population size from the beginning of the nineties until the late nineties, when the stationary phase started, and is still ongoing to this date (Figure 5A,C). Regarding subtype C clade, high exponential growth started in the mid-nineties and was followed by a stationary phase started at the beginning of the 2000s (Figure 5B,D).

**FIGURE 6:**
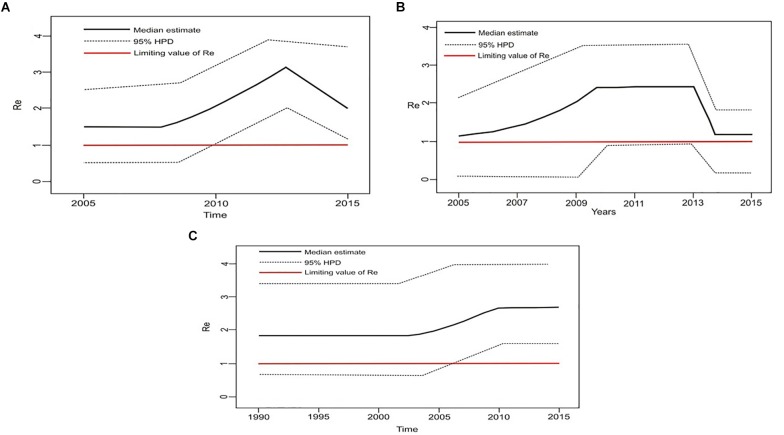
The estimated birth-death skyline serial models by BEAST v2.1 presenting the effective reproductive number (Re) over time for the **(A)** transmission cluster of 15 sequences **(B)** transmission network **(C)** transmission cluster of 11 sequences.

**FIGURE 7:**
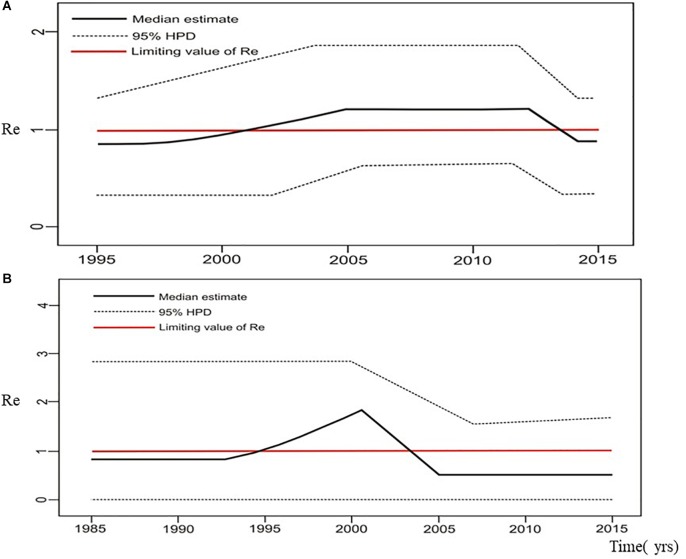
The estimated birth-death skyline serial models by BEAST v2.1. presenting the effective reproductive number (Re) over time for the **(A)** subtype B clade composed of sequences from heterosexuals **(B)** subtype C clade composed of sequences from heterosexuals.

Estimation of the effective reproductive number by birth-death skyline plot analyses showed significant differences among two sub-epidemics in Serbia, the MSM monophyletic clades (Figure 6) and the heterosexual clades (Figure 7). Of note, for all three investigated MSM clades Re over 1 was present during the whole analyzed period (Figure 6A–C). Specifically, the highest Re (maximum value of median Re = 3.2) was found for the most expanded MSM transmission cluster, composed of 15 viral sequences, that also dated to the most recent period (Figure 6A). Similarly, for the second transmission cluster, Re was found to be over 1 during the whole period, reaching the maximum value of 2.3 in 2009 that remained constant for almost 5 years, followed by a decreasing phase, but still retaining value above 1 (Figure 6B). For the transmission network, Re was found to be slightly above 2 for almost 15 years, followed by an increasing phase in 2003, when it reached the value of 2.6 that remained constant approaching the present time (Figure 6C). On the other hand, birth-death skyline plot analyses for heterosexual subtype B monophyletic clade showed Re value below 1, suggesting inactivity for almost 10 years, followed by an active phase when it reached the maximum value of Re in the beginning of the decade of the 2000s, and then a decreasing phase with value below 1 thereafter (Figure 7A). A similar BDM skyline pattern was found for subtype C heterosexual clade, with the obtained Re (median estimates) over 1 for the time period 2003–2012 (Figure 7B).

### Characterizing the Latent Classes

**FIGURE 8:**
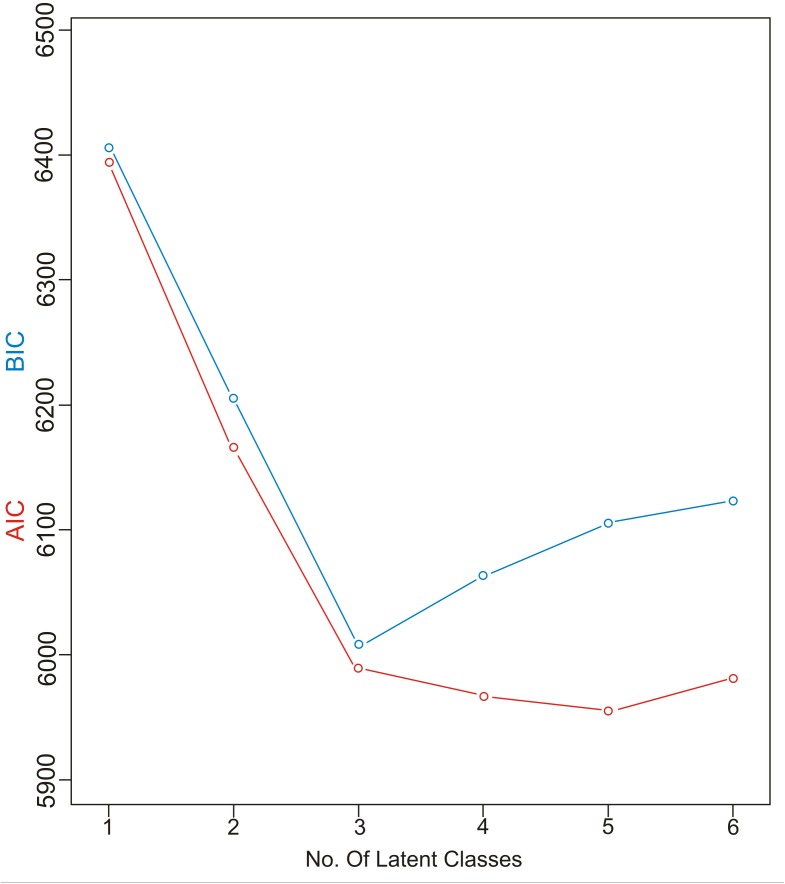
AIC and BIC information criterion values used in model specification.

**FIGURE 9:**
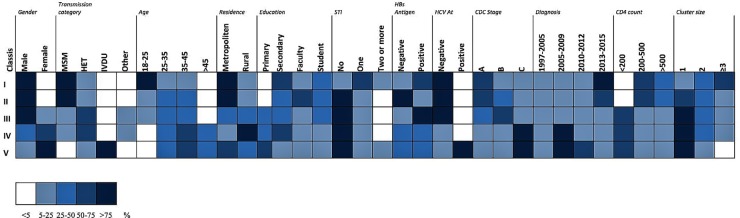
LCA performed in R software with a poLCA software package on data of HIV-1 infected individuals included in the current research. The distribution of participant characteristics within the latent class and predicted proportions in each cluster size by latent class are shown. Darker colors represent higher proportion as shown in the legend.

Akaike information criteria and the BIC values were used as indicated to select the number of latent classes in our LCA model. The best-fitting model calculated in R software in this research suggested five latent classes, and was identified by considering the lowest log likelihood of AIC and BIC values before these values increased with the addition of another class. Figure 8 is a graphic presentation of AIC and BIC numerical data used as statistical support for the number of latent classes. The grouping of patients into five latent classes based on the distribution of different characteristics is shown in Figure 9. Transmission clusters were dominantly present in 2/5 classes. The first class included young MSM subjects aged up to 25 years old, living in Belgrade, with HIV infection diagnosed within the last 3 years of the study period, with 73% of coinfection with HBV and/or other STIs and the highest percentage (53%) of grouping within transmission clusters. The second class encompassed MSM subjects aged up to 45 years of age with high education, HIV diagnosis in the period from the mid-2000s onward, with less than 10% of coinfection with HBV and other STIs and around 20% of sequences found grouped in transmission clusters. In the further three classes no tendency to group in clusters was shown: the third latent class included equal percentages of the MSM transmission group and the heterosexual male subjects, diagnosed during the whole period of the HIV epidemic, from Belgrade, with high percentage of HBV but no other STIs; the fourth latent class encompassed heterosexual subjects of both sexes, aged over 25, with the date of the HIV diagnosis predominantly in the mid-2000s, mostly with secondary education, living in rural areas; the fifth class included female subjects reporting intravenous drug use (IVDU) as a risk for HIV infection, living in Belgrade, with very high percentage of HCV coinfection and diagnosed during whole period of HIV epidemic in Serbia.

## Discussion

Here, we present the study integrating modeling, phylogenetic and socio-demographic analyses of the HIV epidemic in Serbia, to elucidate features, dynamics, and trends of the local sub-epidemics.

The enormous genetic diversity of HIV-1 allows accurate patterns of evolution and transmission to be obtained from samples collected over a relatively short time (Pybus and Rambaut, 2009; Baele et al., 2017). Analysis of phylodynamics makes for a very useful tool for studying molecular epidemiology and aiding public health planning (Pybus and Rambaut, 2009; Baele et al., 2017; Vrancken et al., 2017). If these kinds of sophisticated analyses are based on a multidisciplinary approach, they may give a quantitative description of transmission networks, by identifying socio-demographic correlates of clustering: how virus lineages are restricted to or mix among, different demographic and epidemiological subgroups (Kostaki et al., 2017; Paraskevis et al., 2017).

In the current study, logistic growth modeling of the epidemic in Serbia was performed, based on the registered data on HIV incidence in the period 1984–2016. The obtained model was further used to predict the epidemic trends until 2030. The results for the general and MSM populations in Serbia imply early, exponential phase of HIV epidemic in both cases, with a much steeper slope in the MSM population, potentially providing overall active epidemiological background for transmission clusters to occur. Conversely, in PWID and heterosexual transmission groups, plateaued growth was observed (Supplementary Figures 4, 5). Accurate estimates are important to understand the true burden of HIV, the corresponding need for treatment, and for optimizing testing strategies for HIV. Incidence is the best measure for monitoring infections; however, it is difficult to obtain accurate estimates over time. One of the main approaches to reconstruct the HIV incidence curve is based on the reported number of HIV diagnoses. With the prevalence of HIV infection below 0.1%, Serbia is ranked as a low-prevalence country; however, it is of great importance to study the epidemiological situation in populations at risk such as the MSM and PWID transmission groups and among sex workers. Overall, the highest prevalence of HIV in Serbia has been estimated for MSM (8.3%), much higher when compared to the general population, as well as to other groups at risk such as sex workers (1.8%) and PWID transmission group (1.6%) (UNAIDS, 2017).

A further application of the model for a time period beyond 2017 estimated approaching the plateau in new HIV infections number by 2030 in general population, whereas in MSM transmission group an exponential-like curve with no signs of a plateau in the studied time period was seen. Potential disadvantages of the applied approach include a number of potential biases and difficulties, such as lack of data, underreporting, lack of information on changes over time, etc. However, the obtained results of logistic growth modeling are in line with recent data showing an increase in HIV incidence in Serbia. The incidence in 2000 was 0.02 per 1,000 adults, and in 2016 was 0.05 per 1,000 adults (UNAIDS, 2017). Estimated new HIV infections are decreasing globally, but increasing in the WHO European region, mostly in Eastern and Central Europe (ECDC, 2017; Mravćík et al., 2017). In Serbia, these findings might reflect the fact that though efforts have been made to decentralize preventive and advisory services, the coverage of high-risk groups with preventive services could still be insufficient. This may be due to the lack of recognition of risk behaviors or fear of further stigmatization, especially in smaller communities (Cousins, 2018). In Serbia, a much higher usage of condoms has been reported in professional sex workers than in MSM (UNAIDS, 2017). Moreover, within the MSM population, several vulnerable categories can be found: young men (especially underage boys), MSM involved in sex work (which is illegal), and bisexual men (in particular men who define themselves as heterosexual but who have sex with other men). This latter group may be less likely to be informed than most gay men (Godinho et al., 2005).

The better understanding of genetic history, pattern, and dynamics of HIV transmission on a population level could lead to developments in prevention services, targeting factors associated with onward HIV-1 transmission (Granich et al., 2009; Cohen et al., 2011; Vrancken et al., 2017). In order to determine trends in the HIV epidemic in Serbia, based on HIV-1 pol sequence data generated through the antiretroviral resistance testing, we investigated the transmission clusters and phylodynamic profiles, using in-depth phylogenetic analyses that involve the ML and Bayesian coalescence strategy. These kinds of sophisticated analyses provide an insight into the epidemic trends and patterns of the evolutionary history of a certain viral population, revealing the size of transmission clusters and the dynamics of transmission within them. Interpretation of these results, however, may be hampered if the analyses are not associating other traits with clustering, which serve as determinants of the transmission dynamics and cluster activity (Brenner et al., 2013; Grabowski and Redd, 2014; Chan et al., 2015). Our previous study established the role of transmission clusters/networks in HIV epidemic spread in Serbia, however, phylodynamic aspects were not considered nor the impact of different socio-demographic and clinical characteristics on the clustering patterns (Siljic et al., 2013). In the current study, we found a high percentage of 53% of sequences involved in transmission clusters/network in Serbia. This finding is in line and even higher than our previous findings and studies of other epidemics (Leigh Brown et al., 2011; Siljic et al., 2013; Ragonnet-Cronin et al., 2016; Chaillon et al., 2017; Parczewski et al., 2017). Notably, it is considered that assessment of HIV transmission clusters is influenced by sampling density – the higher the sampling density, the more important the clustering (Novitsky et al., 2014). However, methods and definitions of transmission clusters in HIV phylogenies are still hotly debated – recent findings suggest that bootstrap support should be replaced or complemented by newer methods, such as refined, gradual function in large data sets (Lemoine et al., 2018). In view of the analyzed sample size we maintained a more conservative approach by using both bootstrap and Bayesian posterior probability, as the latter provides closer estimates of the true probabilities of recovering clades (Wilcox et al., 2002). Again, having in mind the studied sample size, the obtained, high enough total percentage of 53% of clustered sequences, may be even considered as a rather conservative, lower edge estimate. Our results suggest that HIV spread is driven by local MSM transmission, as observed also in other European countries (Sullivan et al., 2009; van Griensven et al., 2009; Frentz et al., 2013; Phillips et al., 2013). Similar to some other European countries, clustering sequences from MSM were found within subtype B (Abecasis et al., 2013; Esbjörnsson et al., 2016). Furthermore, we found an extension of previously reported small transmission clusters related to young newly diagnosed MSM patients (Siljic et al., 2013). Most of the heterosexual transmissions seemed to be limited to transmission pairs and small clusters, without substantial further spread of the infection. All things considered, our results show that local HIV transmission in Serbia is mainly driven by MSM transmission clusters.

Based on the effective reproductive number (Re) estimated through birth-death plots, together with the number of infections over time, significant differences between the MSM and heterosexual clades were found. MSM subtype B clades showed mean reproductive number over one during the whole investigated period, with the steepest slopes and a recent tMRCA, in line with a growing trend in the number of transmissions in years approaching the end of the study period. On the contrary, heterosexual clades in both studied subtypes – B and C, showed modest growth and stagnation. This finding could be influenced by the analyzed sample size, with a possible underestimation and bias toward being slower/lower at the moment compared to the MSM route but with possibility of change. However, neither epidemiological data and trends nor other analytical approaches (such as LCA analysis) imply that changes in the sub epidemic dynamics would be plausible.

The obtained results imply that none of the clusters will stop to be active in the near future as most clusters contain recent infections and are rejuvenated by the inclusion of younger men, and many have a reproduction number greater than the epidemic threshold. Although the transmission network was defined to be active, with reproductive potential striking higher than epidemic threshold and increasing in later years, it also appears to be “aging,” while clusters appear to be of lower mean age; especially the most expanded cluster that was found to be “the youngest” was also found to be the one with the highest reproductive potential. On the other hand, particularly worrying is the fact that even though the transmission network comprised sequences from MSM diagnosed early in infection, this did not seem to have stopped clusters from growing. Other researchers showed that a large part of onward transmission amongst MSM is related to the early phase of infection (Bezemer et al., 2010, 2015;Powers et al., 2011).

HIV transmission dynamics is considered to be shaped by a number of diverse constraints within the host, upon a transmission process and at the population level, influencing viral evolution (within-host and inter-host), virus and host genetics, complex interplay of between-host interactions but also important social and demographic factors (Theys et al., 2018). Hence, the inference of HIV-1 transmission dynamics and factors influencing epidemic spread may provide an important input for the design of efficient public health interventions, and new approaches are constantly being explored (Poon, 2016; Le Vu et al., 2018). The LCA analysis provided further insights by combining clustering patterns and a set of characteristics associated with patients such as socio-demographic, clinical, transmission risk, diagnosis date. Together, these features allow assessing multiple determinants of the local HIV transmission, helping to understand its occurrence in a “real life” manner. This analysis has separated five subgroups (latent classes) with various combinations of analyzed characteristics that affect differently the epidemic in Serbia. Importantly, four factors were found to be significantly associated with patients belonging to clusters: very young age, residing in the capital city, recent diagnosis and a high rate of STI and HBV coinfections.

According to the latest European epidemiological data, sex between men remains the predominant mode of HIV transmission and MSM are disproportionately at risk for and affected by HIV, STIs and viral hepatitis (ECDC, 2015, 2017, 2018). A similar situation has been increasingly described in different regions worldwide (Qi et al., 2015). Current WHO guidelines recommend oral pre-exposure prophylaxis (PrEP) to be offered as an additional prevention choice for people at substantial risk of HIV infection as part of a combination of HIV prevention approaches, where ‘substantial risk’ of HIV infection is provisionally defined as HIV incidence greater than 3 per 100 person-years (WHO, 2015). In Serbia, WHO 90–90–90 target has been embraced, however, PrEP is not yet available. Monitoring the HIV epidemic is essential for assessing the impact of effective HIV prevention interventions, determining public health priorities, and estimating current and future health care needs.

In summary, the results presented imply that the HIV epidemic in Serbia is still in the exponential growth phase, in particular, related to the MSM transmission that is estimated to retain the steep growth curve until 2030. The previously described tendency of cluster formation in this group has been confirmed. The obtained results imply that an average new HIV patient in Serbia is a young man with concomitant STIs. Together, these findings provide a useful insight that may prove to be vital for prospective public health priorities and interventions, in particular, relative to the 90–90–90 targets and considerations of PrEP.

## Ethics Statement

This study was carried out in accordance with the recommendations of University of Belgrade Faculty of Medicine Ethical Committee with written informed consent from all subjects. All subjects gave written informed consent in accordance with the Declaration of Helsinki. The protocol was approved by the University of Belgrade Faculty of Medicine Ethical Committee, decision No 29/V-11.

## Author Contributions

MS, LJ, and MŠ conceived and designed the study. MŠ, VĆ, and MT performed molecular analyses. DS, IP-P, JR, and DJ collected and analyzed the epidemiological data. LJ performed modeling analysis. LJ, MŠ, and VĆ performed phylogenetic analyses. MS, LJ, and MŠ have been involved in drafting the manuscript. All authors read and approved the final manuscript.

## Conflict of Interest Statement

The authors declare that the research was conducted in the absence of any commercial or financial relationships that could be construed as a potential conflict of interest.

## Footnotes

1 http://www.batut.org.rs/index.php?category_id=17

2 http://www.nlreg.com

3 https://www.hiv.lanl.gov/content/index

4 http://dbpartners.stanford.edu:8080/RegaSubtyping/stanford-hiv/typingtool/

5 www.hiv.lanl.gov

6 https://hivdb.stanford.edu/hivdb/by-mutations/

7 https://www.megasoftware.net/

8 http://tree.bio.ed.ac.uk/software/figtree/

9 http://blast.ncbi.nlm.nih.gov

10 http://tree.bio.ed.ac.uk/software/tracer/

11 http://beast.bio.ed.ac.uk/Tracer

12 http://tree.bio.ed.ac.uk/software/tracer/

13 http://CRAN.R-project.org/package=poLCA
