# Supplementary material for: Exploring Evolutionary and Transmission Dynamics of HIV Epidemic in Serbia: Bridging Socio-Demographic With Phylogenetic Approach
Source: Front Microbiol. 2019 Feb 25;10:287. doi: 10.3389/fmicb.2019.00287 (PMC6397891; doi:10.3389/fmicb.2019.00287)
Supplement: Supplementary file 2 [file Table_3.DOCX]

Supplementary data

In order to compare and contrast two main sub-epidemics in Serbia, MSM and heterosexual, three MSM clades, accomplishing predefined phylogenetic criteria and two heterosexual clades, without enough support to be considered as transmission clusters were analyzed by means of phylodynamic analysis. To verify phylodynamic analysis performed on these heterosexual clades one additional MSM clade supported by lower bootstrap value than predefined, was analyzed. This phylodynamic analysis was performed by setting the same conditions as described in the manuscript. Bayesian skyline plot analysis, used to infer the estimation of the effective population size over time, identified relatively stable growth phase till the end of 2003 followed by high exponential growth phase with slight decline in a growth rate in 2009 and again slight increase in 2012 (Supplementary figure 1). Logistic growth model suggested high exponential growth phase followed by stable growth phase and slight increase phase in the end of analyzed period (Supplementary figure 2). Estimation of the effective reproductive number, by birth-death skyline plot analyses, showed Re higher than 1 through all analyzed period with high increasing phase started around 2009 reaching maximum value of 2 in 2013, that was followed by decreasing phase (Supplementary figure 3).

Furthermore growth trends of heterosexual transmission group as well as PWID transmission group were made in order to assess potential confounding in growth pattern of national HIV epidemic (Supplementary figures 4 and 5). The analysis showed less steep growth curve of heterosexul and PWID transsmision compared with MSM transmission growth with evident plateau by the year 2030. This findings complement phylodynamic characteristics of the MSM clades observed.

Supplementary figures text

Supplementary figure 1.

Bayesian skyline reconstruction analysis performed for the sequences within MSM clade supported by lower bootstrap than predefined.

Supplementary figure 2.

Logistic growth analysis performed in BEAST v1.7.5 for the sequences within MSM clade supported by lower bootstrap than predefined.

Supplementary figure 3.

The estimated birth-death skyline serial models by BEAST v2.1. presenting the effective reproductive number (Re) over time for the sequences within MSM clade supported by lower bootstrap than predefined.

Supplementary figure 4.

Logistic growth model of new HIV infections in PWID transmission group in Serbia in the period 2003-2030. On y-axis cumulative number of new HIV cases is shown, and on the x time period in years is shown.

Supplementary figure 5.

Logistic growth model of new HIV infections in heterosexual transmission group in Serbia in the period 2003-2030. On y-axis cumulative number of new HIV cases is shown, and on the x time period in years is shown.
