# Supplementary material for: Exploring Evolutionary and Transmission Dynamics of HIV Epidemic in Serbia: Bridging Socio-Demographic With Phylogenetic Approach
Source: Front Microbiol. 2019 Feb 25;10:287. doi: 10.3389/fmicb.2019.00287 (PMC6397891; doi:10.3389/fmicb.2019.00287)
Supplement: Supplementary file 4 [file Data_Sheet_2.docx]

**Supplementary data file**

1. **Accession numbers of Serbian sequences included in the present study, sampled from 1997 to 2015 and deposited in the NCBI database:**

GQ399763.1, GQ399551.1, GQ400327.1, GQ400482.1, GQ398972.1, GQ399179.1, GQ399012.1, GQ399888.1, GQ399221.1, GQ399018.1, GQ480327.1, GQ399263.1, GQ395533.1, GQ400459.1, GQ400490.1, GQ400092.1, GQ399955.1, GQ400505.1, GQ399341.1, GQ399810.1, GQ398855.1, GQ400529.1, GQ400303.1, GQ400203.1, GQ400169.1, GQ399328.1, GQ398698.1, GQ399770.1, GQ399505.1, GQ399605.1, GQ399463.1, GQ399262.1, GQ400380.1, GQ399684.1, GQ399526.1, GQ400192.1, GQ399335.1, GQ400562.1, GQ400664.1, GQ400943.1, GQ400568.1, GQ400867.1, GQ399293.1, GQ400985.1, GQ400636.1, GQ400934.1, GQ400637.1, GQ400971.1, GQ400727.1, GQ400860.1, GQ400842.1, GQ400711.1, GQ400847.1, GQ400975.1, GQ401005.1, GQ400863.1, GQ399151.1, GQ400634.1, GQ400696.1, GQ400623.1, GQ400576.1, GQ400698.1, GQ400683.1, JX299860.1, JX300595.1, JX300466.1, JX301157.1, JX300670.1, JX300934.1, JX300963.1, JX301026.1, JX299883.1, JX299967.1, JX300342.1, JX300698.1, JX301113.1, JX299941.1, JX300732.1, KF056325,  MH750236.1,  MH750235.1,

KF157408 - KF157549

 MK253347-MK253439

1. **Accession numbers of reference sequences used for phylogenetic analysis were as follows:**

Subtype A (AF069670); B (K03455); C (U52953); G (AF061642); CRF01_AE (U54771); CRF02_AG (AF063223); F (AJ249236, AF075703, AF07733, AF377956).

1. **HIV-1 control/background sequences sampled across Europe, Northern America and Africa downloaded from the NCBI database (**[**http://www.ncbi**](http://www.ncbi)**. nlm. nih.gov/nuccore) used for the phylogenetic analysis for both the second and the third dataset:**

Albania: AY611666; AY611672; AY611684; AY611688

Austria : AF347214; AF347518; DQ878531; DQ878532; EJ936557

Belgium: DQ177230; DQ177232; DQ877759; FJ653084; EU248460; DQ177224; DQ177231; DQ177227

Bulgaria: EF517439; EF517457; EF517462; EF517464; EF517488; EF517472; EF517439; EF517410

Croatia: FN424300; FN424301

Cyprus : EU673375; EU673382; EU673408

Czech Republic: AY694218; AY694233; AY694364

Denmark: AJ419453; AJ582147; AM490879; DQ108366; DQ877795

France: AF487122; DQ878075; DQ877953; DQ877930

Germany: AF347190; AF347288; AF347140; AY878668; AY878677; DQ878276; DQ878304 ; FJ030769; GQ400800

Greece: DQ878544; DQ878548; DQ878559; DQ878569; DQ878595; EF563173

Greenland: AM285220; AM285242; AM285267; AM937019; AM937024

Ireland : DQ877830; DQ877832;

Italy: AY375051; AY362443; DQ348057; DQ348033; DQ345139; DQ345123; DQ345246; DQ345123; DQ345262; DQ345233; DQ345221; AF251947; AF252026; AF376547; AF493371; AF517266; AF517471; AY672455; AY352444; AY855419; AY855724; AY994341; AY995503; DQ345170; DQ345265; DQ369253; DQ672623; DQ878603; EF526205; EU019810; EU496146; FJ228037; FJ228081; FJ228038; FJ209055; FJ209061; FJ228131; FJ228123; FJ228127

Luxembourg: DQ877749; EF563190

Netherlands: AY423387; AY423383; AY877314; DQ877839; U34604; GQ399672; DQ877848
