# Supplementary figures and images for: Exploring Evolutionary and Transmission Dynamics of HIV Epidemic in Serbia: Bridging Socio-Demographic With Phylogenetic Approach
Source: Front Microbiol. 2019 Feb 25;10:287. doi: 10.3389/fmicb.2019.00287 (PMC6397891; doi:10.3389/fmicb.2019.00287)

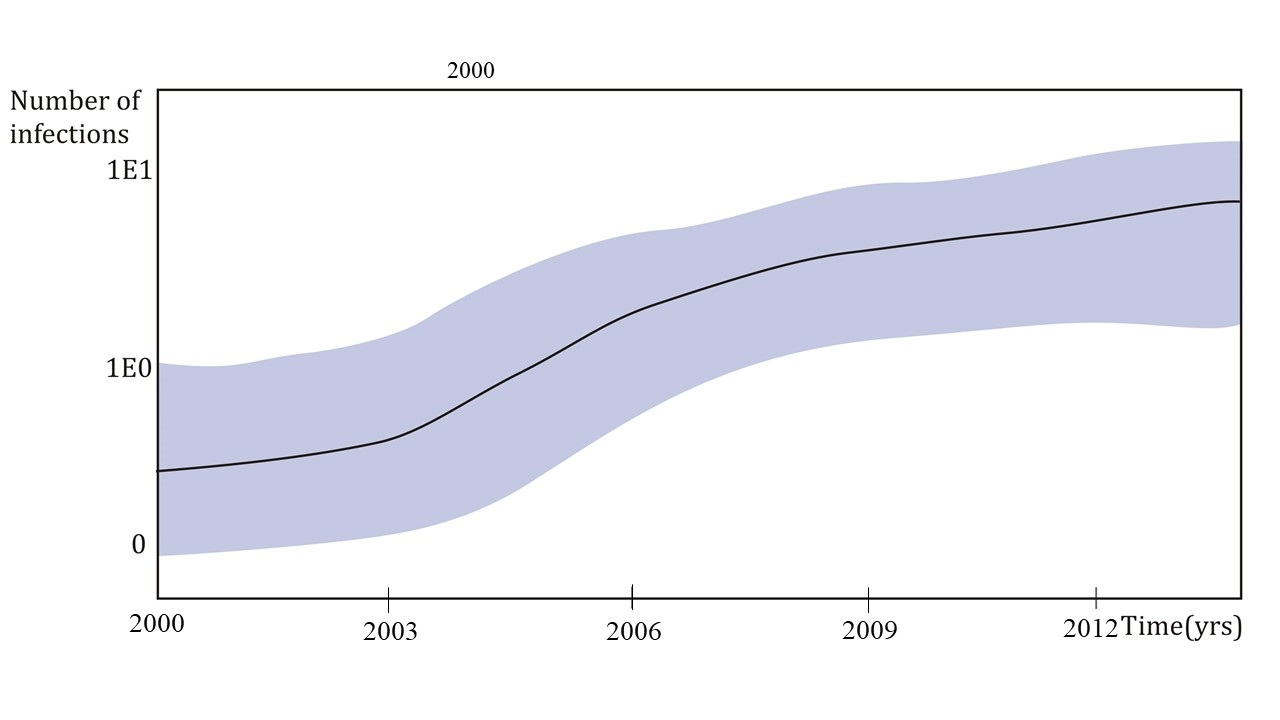

Supplement: Supplementary file 5 [file Image_1.TIF]

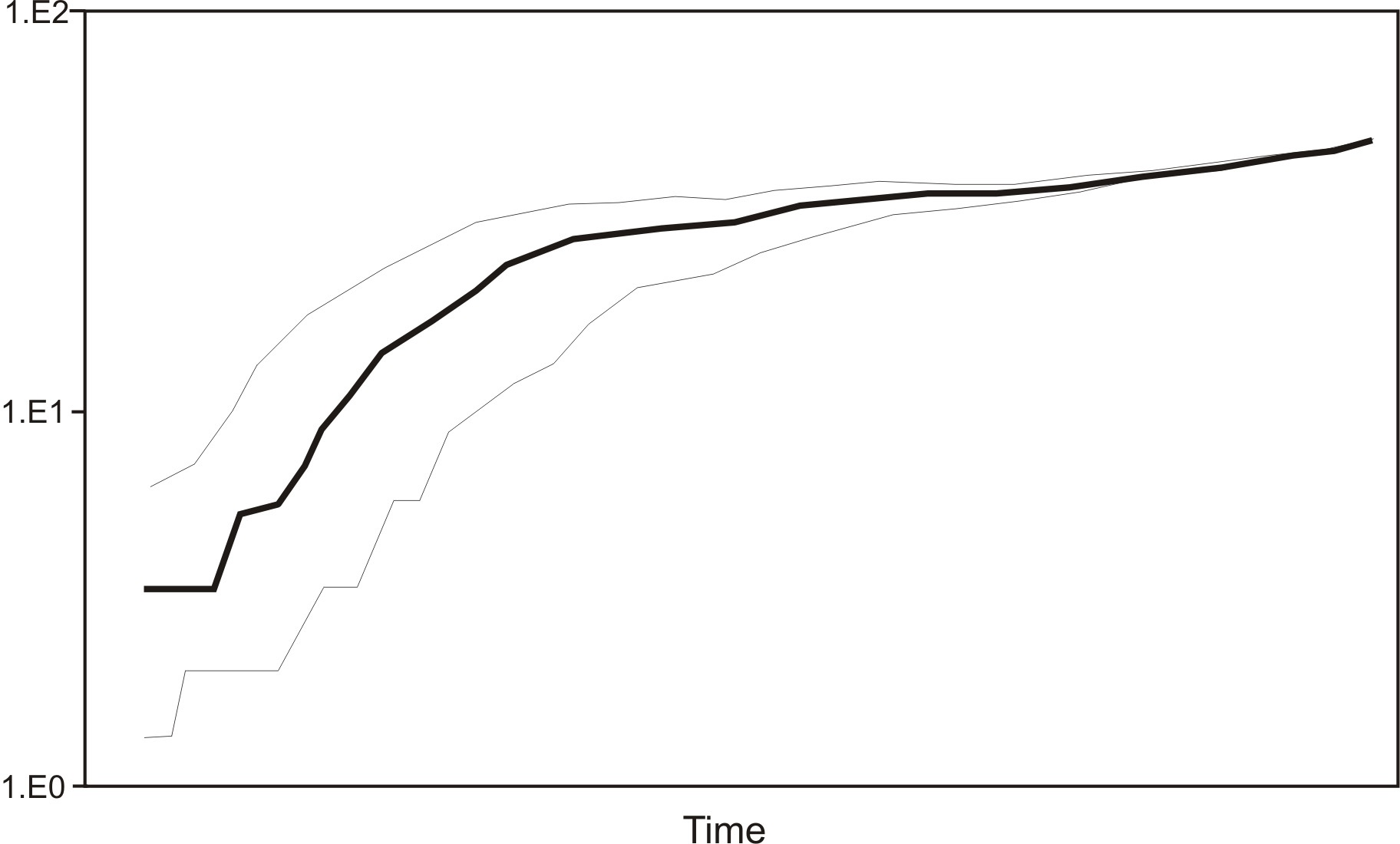

Supplement: Supplementary file 6 [file Image_2.TIF]

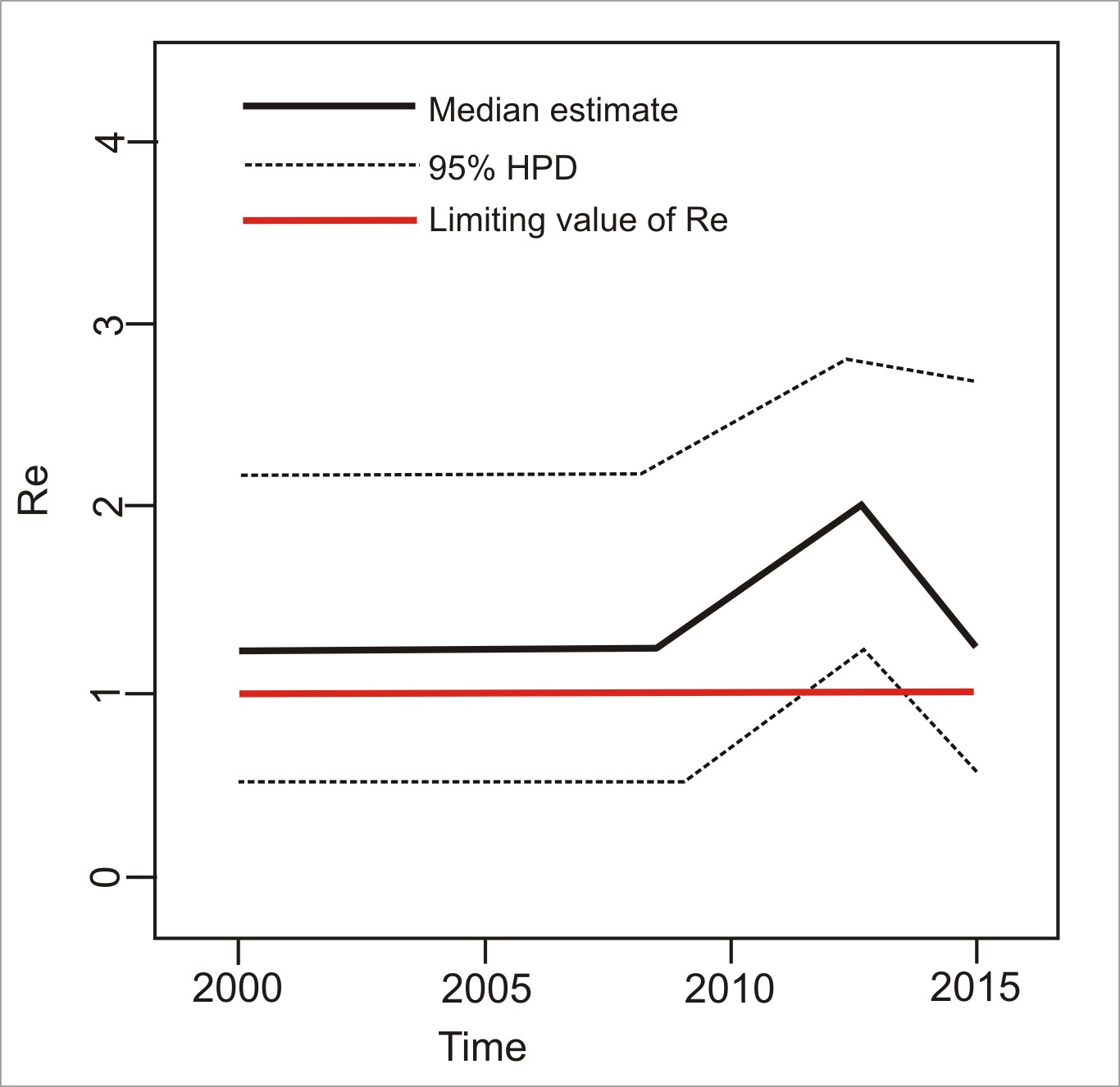

Supplement: Supplementary file 7 [file Image_3.TIF]

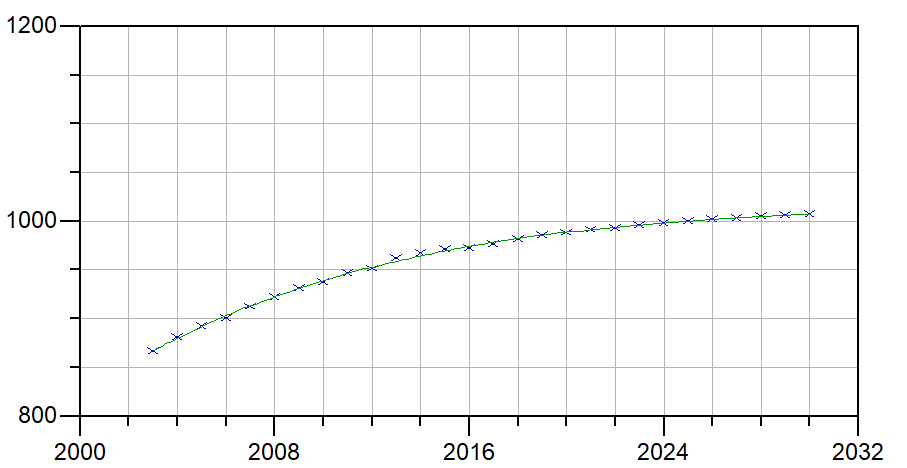

Supplement: Supplementary file 8 [file Image_4.JPEG]

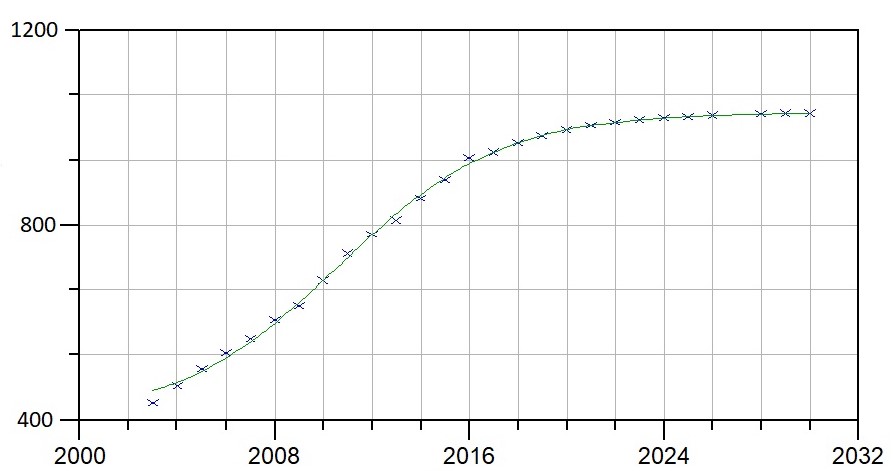

Supplement: Supplementary file 9 [file Image_5.JPEG]
